# Early Attrition Prediction for Web-Based Interpretation Bias Modification to Reduce Anxious Thinking: A Machine Learning Study

**DOI:** 10.2196/51567

**Published:** 2024-12-20

**Authors:** Sonia Baee, Jeremy W Eberle, Anna N Baglione, Tyler Spears, Elijah Lewis, Hongning Wang, Daniel H Funk, Bethany Teachman, Laura E Barnes

**Affiliations:** 1 Department of Systems and Information Engineering University of Virginia Charlottesville, VA United States; 2 Department of Psychology University of Virginia Charlottesville, VA United States; 3 Department of Electrical and Computer Engineering University of Virginia Charlottesville, VA United States; 4 Department of Computer Science University of Virginia Charlottesville, VA United States; 5 Department of Computer Science and Technology Tsinghua University Beijing China; 6 Sartography Staunton, VA United States

**Keywords:** digital mental health intervention, attrition prediction, user engagement, cognitive bias modification, CBM-I, dropout rate, personalization

## Abstract

**Background:**

Digital mental health is a promising paradigm for individualized, patient-driven health care. For example, cognitive bias modification programs that target interpretation biases (cognitive bias modification for interpretation [CBM-I]) can provide practice thinking about ambiguous situations in less threatening ways on the web without requiring a therapist. However, digital mental health interventions, including CBM-I, are often plagued with lack of sustained engagement and high attrition rates. New attrition detection and mitigation strategies are needed to improve these interventions.

**Objective:**

This paper aims to identify participants at a high risk of dropout during the early stages of 3 web-based trials of multisession CBM-I and to investigate which self-reported and passively detected feature sets computed from the participants interacting with the intervention and assessments were most informative in making this prediction.

**Methods:**

The participants analyzed in this paper were community adults with traits such as anxiety or negative thinking about the future (Study 1: n=252, Study 2: n=326, Study 3: n=699) who had been assigned to CBM-I conditions in 3 efficacy-effectiveness trials on our team’s public research website. To identify participants at a high risk of dropout, we created 4 unique feature sets: self-reported baseline user characteristics (eg, demographics), self-reported user context and reactions to the program (eg, state affect), self-reported user clinical functioning (eg, mental health symptoms), and passively detected user behavior on the website (eg, time spent on a web page of CBM-I training exercises, time of day during which the exercises were completed, latency of completing the assessments, and type of device used). Then, we investigated the feature sets as potential predictors of which participants were at high risk of not starting the second training session of a given program using well-known machine learning algorithms.

**Results:**

The extreme gradient boosting algorithm performed the best and identified participants at high risk with macro–*F*_1_-scores of .832 (Study 1 with 146 features), .770 (Study 2 with 87 features), and .917 (Study 3 with 127 features). Features involving passive detection of user behavior contributed the most to the prediction relative to other features. The mean Gini importance scores for the passive features were as follows: .033 (95% CI .019-.047) in Study 1; .029 (95% CI .023-.035) in Study 2; and .045 (95% CI .039-.051) in Study 3. However, using all features extracted from a given study led to the best predictive performance.

**Conclusions:**

These results suggest that using passive indicators of user behavior, alongside self-reported measures, can improve the accuracy of prediction of participants at a high risk of dropout early during multisession CBM-I programs. Furthermore, our analyses highlight the challenge of generalizability in digital health intervention studies and the need for more personalized attrition prevention strategies.

## Introduction

### Background

Approximately half of the US population experience a mental illness during their lifetime [1,2]. During the early stage of the COVID-19 pandemic, researchers estimated an increase of 25.6% in new cases of anxiety disorders per 100,000 people globally [3]. Mental illness is associated with impaired daily functioning, more frequent use of health care resources, and increased risk of suicide [1]. However, more than two-thirds of individuals with a mental illness do not receive treatment [4]. A multitude of barriers impede the initiation and sustained use of face-to-face (ie, traditionally delivered) treatment, including stigma; cost; lack of insurance coverage; and limited availability of support services, especially trained clinicians [2,5-7]. Given these challenges, there is an urgent need to help people manage their mental health in new ways [1,5].

Digital mental health interventions (DMHIs), which harness digital technologies to promote behavior change and maintain health [8], provide an appealing alternative for much-needed treatment outside a clinician’s office [9]. DMHIs may help individuals overcome obstacles to treatment, such as geographic or financial constraints, and may thus reduce the treatment gap among the broader population. Given the limited resources for health care service delivery, low-cost mobile health and eHealth interventions could be key to supporting symptom monitoring and long-term self-management of patients with mental disorders [10]. With an increasing demand for mental health care amid a shortage of mental health professionals, the use of eHealth and mobile health apps is expanding [11-13]. While these solutions have the potential to play an important role in increasing access to mental health services, especially for underserved communities, the clinical community is still determining how to best leverage these solutions [14].

Poor adherence and substantial dropout are common challenges in DMHIs [15]. *Adherence*, the extent to which users complete a DMHI’s tasks as intended [16,17], is likely to be associated with better treatment outcomes [1]. Although these tasks can vary widely (given the varied designs of DMHIs [16]), it is through engaging with such tasks that DMHIs are thought to achieve their outcomes [17]. However, sustained engagement with these platforms remains a significant issue [10,18-22]. Digital health interventions suffer from rates of dropout ranging from 30% to as high as 90% [1,5,15,19,23,24]. *Dropout* occurs when a participant prematurely discontinues an intervention (due to various potential reasons, such as technical issues, lack of time or energy, and lack of perceived benefit [18]). Even a modest dropout rate can limit the generalizability of digital intervention findings to only those who completed the study; thus, effective evaluation of treatments becomes a challenge [10,15,21,25-27]. This likely contributes to the uncertainties among clinicians and patients regarding the efficacy, usability, and quality of DMHIs [10]. There are many reasons clinicians tend not to integrate DMHIs into their clinical practice (eg, insufficient knowledge about DMHIs and lack of training about how to integrate them [28,29]). An additional reason is that if patients’ sustained engagement with DMHIs is low and they stop participating in the intervention before achieving meaningful gains, then clinicians have little incentive to view DMHIs as a helpful tool to increase the efficiency and impact of care.

One approach to reducing attrition in DMHIs is to identify participants at a high risk of dropping out at the early stages of the intervention, which would permit the intervention to be adapted to these users’ needs [30]. For example, more support (eg, minimal human contact with a telecoach) could be offered specifically to such users (thereby maintaining scalability [31]). Although increasing attention has recently been dedicated to attrition in various eHealth interventions [32-34], relatively few advances within DMHIs have predicted dropout through streamlined quantitative approaches considering both passive and self-reported data. Testing the effectiveness of interventions on treatment outcomes [35] often takes priority rather than identifying and predicting users at high risk of attrition. Consequently, methodological advancements in attrition prediction have largely taken place outside clinically relevant settings, such as in the eCommerce and social gaming industries [36-38]. This paper develops a data-driven algorithm that includes both passive indicators of user behavior and self-reported measures to identify individuals at a high risk of early attrition in 3 DMHIs; as such, it provides a framework that helps in the personalization of DMHIs to suit individual users based on each individual’s attrition risk.

To predict attrition in DMHIs, there are 2 main considerations [18]. First, we need to define the prediction horizon; that is, researchers should determine the point in an intervention’s timeline at which it would be beneficial to predict which participants are at a high risk of dropping out. This decision may be influenced by an analysis of when in the timeline most participants are actually dropping out; such an analysis may allow the identification and strengthening of weak parts of an intervention. Given that low engagement has been consistently cited as the construct underlying attrition, this decision may also be informed by considering typical patterns of engagement [10,36,37,39-43]. However, engagement is a very broad construct with many components [17], and empirical evidence suggests that engagement fluctuates with time [30]. Thus, carefully defining the feature space and predicting participants who are at a high risk of attrition at meaningful time points in a program can provide valuable information. For example, participants may initially stay in the intervention out of curiosity, which relates to the novelty effect—the human tendency to engage with a novel phenomenon [35], but then lose interest. If a researcher wants to mitigate the impact of the novelty effect, then understanding early-stage dropout (ie, early in the program but after it is no longer brand new and unknown) is critical.

Second, we must consider which factors cause users to drop out of a given DMHI. Answering this question can help researchers and designers tailor the intervention to particular user groups. Demographic variables such as gender, age, income, and educational background have been related to higher attrition rates in digital health interventions [10,44-47]. With respect to participants’ mental health (eg, lifetime symptoms assessed at baseline or current symptoms assessed during the course of the intervention), the presence of mental health symptoms may increase interest toward the use of a digital intervention in an effort to reduce such symptoms [26]. However, certain symptoms (eg, hopelessness) may reduce the participants’ motivation or ability to sustain engagement with an intervention [10,15,20,22,33,48]. In addition to these baseline user characteristics, user clinical functioning (ie, current symptoms and psychological processes that lead to the maintenance of these symptoms), self-reported user context and reactions to interventions (eg, perceived credibility of DMHIs, which is associated with increased engagement and reduced dropout [10]), and passively detected user behavior influence attrition rates in digital platforms [15,31]. This behavior includes time spent using an intervention [38,49,50], the passively detected context (eg, time of the day and day of the week) [49], and type of technology (eg, web, smartphone, computer based, or wearable) [20,51].

Prior studies, mainly in psychology, have predicted attrition primarily with statistical techniques such as ANOVA and regression [46,47,52-54]. In addition, other research has used macrolevel approaches, such as contrasting one intervention’s attrition rate against another’s [39] and examining participant and psychotherapy trial factors that predict dropout rates [55]. Researchers in computer and data science and the mobile gaming industry more commonly leverage passively collected behavioral data from users and have found success in predicting attrition (“churn”) using more advanced techniques, such as linear mixed modeling [37], survival analysis [38], and probabilistic latent variable modeling [36]. More recently, advanced machine learning models, such as deep neural networks, have also been useful for modeling and predicting attrition in mobile gaming [38,50,56,57] and in digital health care applications [20,58]. Our approach builds on work predicting attrition in DMHIs [37,45,47,54,58-60] and incorporates both passively collected behavioral data and self-reported data [1,17,31,60-63].

An attractive DMHI for anxiety is cognitive bias modification for interpretation (CBM-I [64,65]), a web-based program with potential to reach large, geographically diverse samples of adults with anxiety symptoms. CBM-I aims to shift threat-focused interpretation biases in which people with anxiety symptoms tend to assign a negative or catastrophic meaning to situations that are ambiguous. Cognitive models of anxiety suggest that training people with anxiety symptoms to consider benign interpretations of ambiguous situations, as opposed to only rigidly negative interpretations, may reduce anxiety [66-68]. To shift interpretation biases, CBM-I training sessions prompt users to imagine themselves in ambiguous, threat-relevant scenarios (presented in a set of short sentences) and to practice disambiguating each scenario by filling in its final word (typically presented as a word fragment) [65]. Active CBM-I conditions encourage more positive and flexible interpretation of scenarios by providing a final word that assigns a benign or a positive meaning to the ambiguous situation (consider this example: “As you are walking down a crowded street, you see your neighbor on the other side. You call out, but she does not answer you. Standing there in the street, you think that this must be because she was distracted.”). By presenting benign or positive endings for most scenarios (eg, 90%), positive CBM-I conditions train a positive contingency in which users learn to expect that ambiguous potentially threatening situations usually work out fine.

The greatest degree of improvement is expected in positive conditions relative to other active conditions (eg, 50% positive and 50% negative conditions that present positive and negative endings in equal proportions, thereby training flexible interpretation but no contingency) and to control conditions (eg, no training or a neutral condition with emotionally unambiguous scenarios and neutral endings). Thus, this paper focuses on attrition in positive conditions. Despite some mixed results [62,69], a number of studies have shown the effectiveness of positive CBM-I conditions in shifting interpretation biases and reducing anxiety symptoms [19,44,64,70-72]. To benefit from CBM-I programs, people must be able to use them effectively during a sustained period. However, similar to many DMHIs, web-based CBM-I programs face substantial attrition rates [19,73].

### Objective

This paper has 3 aims. The first aim is to determine a practical attrition prediction horizon (ie, to determine the session at which it would be beneficial to identify individuals at a high risk of dropping out). The second aim is to identify participants at a high risk of dropping out by leveraging baseline user characteristics, self-reported user context and reactions to the program, passively detected user behavior, and clinical functioning of users within our analysis. The third aim is to explore which of these feature sets are most important for the identification of participants at high risk. To achieve these aims, we propose a multistage pipeline to identify participants who are at a high risk of dropout from the early stages of 3 different DMHI studies. These interventions use web-based CBM-I [64,65] to help individuals change their thinking in response to situations that make them feel anxious or upset [19,44,74]. Note that our proposed pipeline is expected to apply broadly to DMHIs; however, in this paper, we focus on CBM-I programs as a useful starting point and look for important features of attrition in such programs.

## Methods

### Data Source and Interventions

MindTrails [75] is a multisession, internet-delivered CBM-I training program. To date, >6000 people across >80 countries have enrolled in MindTrails, pointing to participant interest in accessing a technology-delivered, highly scalable intervention that can shift anxious thinking in a targeted and efficient way.

In this paper, we focus on 3 MindTrails studies: Managing Anxiety, Future Thinking, and Calm Thinking. We provide a brief overview of these studies, which were approved by the University of Virginia Institutional Review Board (IRB). We analyzed data from 1277 participants who took part in these studies. Details of the studies are provided in Table 1.

**Table 1:** Overview of MindTrails studies.

Study name	Duration	Target population	Number of CBM-I^a^ training sessions	Valid participants in parent study, n	Positive CBM-I participants^b^, n	Engagement strategy	
						Compensation	Session reminder
Managing Anxiety	Jun 8, 2016, to January 20, 2019	Adults with anxiety	8	807	252	None	Emails
Future Thinking	May 3, 2017, to October 16, 2019	Adults with negative expectations about the future	4	1221	326	None	Emails, text messages
Calm Thinking	May 18, 2019, to November 13, 2020	Adults with anxiety	5	1748	699	US $25^c^	Emails, text messages

^a^CBM-I: cognitive bias modification for interpretation.

^b^Condition of interest for this paper’s analyses.

^c^US $5 per assessment at baseline, after Session 3, and after Session 5; US $10 for follow-up assessment.

### Participants and Procedure

#### Study 1: Managing Anxiety

The Managing Anxiety study focused on the development of an infrastructure to assess the feasibility, target engagement, and outcomes of a free, multisession, web-based CBM-I program for anxiety symptoms. A large sample of community adults with at least moderate trait anxiety based on an anxiety screener (Anxiety Scale of the 21-item Depression Anxiety Stress Scales, DASS-21 [76]) was randomly assigned to (1) positive CBM-I training (90% positive and 10% negative), (2) 50% positive and 50% negative CBM-I training, or (3) a no-training control condition. Toward the start of CBM-I training, participants also underwent an imagery prime manipulation, an imagination exercise designed to activate the participants’ anxious thinking about a situation in their life. After consenting and enrolling, the participants completed a battery of baseline measures, including demographic information, mental health history, and treatment history. For details about the Managing Anxiety study protocol, including the aims and the outcome measures of the study, refer to the main outcomes paper by Ji et al [19].

The program involved up to 8 web-based training sessions, delivered at least 48 hours apart, with assessments immediately after each session and a follow-up assessment 2 months after the last session. During each session, CBM-I training was provided. This training involved 40 training scenarios, which were designed to take approximately 15 minutes to complete. Study contact, in the form of automated reminder emails sent to all participants, was equivalent in content and schedule regardless of training condition. If participants completed only part of an assessment task, they continued the assessment the next time they returned. If they completed only part of a training task, they restarted the task upon returning. Participants received no monetary compensation. A total of 3960 participants completed the eligibility screener, out of which 807 (20.38%) eligible participants enrolled and completed the baseline assessment. In this paper, only data from the positive intervention arm (ie, positive CBM-I condition) were used (n=252, 31.23% of participants who enrolled and completed the baseline assessment), given our interest in testing predictors of attrition in positive CBM-I across all 3 studies.

#### Study 2: Future Thinking

The Future Thinking study, a hybrid efficacy-effectiveness trial, focused on testing a multisession, scalable, web-based adaptation of CBM-I to encourage healthier, more positive future thinking in community adults with negative expectations about the future based on the Expectancy Bias Task (shortened from the version used by Namaky et al [77]). After completing the screener, eligible participants provided consent; were enrolled; and were randomly assigned to (1) positive conditions with ambiguous future scenarios that ended positively, (2) 50-50 conditions that ended positively or negatively, or (3) a control condition with neutral scenarios. For details about the aims and outcome measures of the Future Thinking study, refer to the main outcomes paper by Eberle et al [44].

The participants were asked to complete 4 training sessions (40 scenarios each). Assessments were given at baseline, immediately after each session, and during the follow-up assessment 1 month after the last session. Participants had to wait for 2 days before starting the next training session; they had to wait for 30 days before starting the follow-up assessment. Participants had the option of receiving an email or SMS text message reminder when the next session or follow-up assessment was due. If they completed only part of a training or assessment task, they continued the task the next time they returned. The participants received no monetary compensation. A total of 4751 participants completed the eligibility screener, out of which 1221 (25.70%) were eligible and were enrolled. In this paper, only data from the positive CBM-I intervention arm (ie, the positive condition and the positive + negation condition) were used (n=326, 26.70% of enrolled participants).

#### Study 3: Calm Thinking

The Calm Thinking study, a sequential, multiple assignment, randomized trial, tested the effectiveness of positive CBM-I relative to a psychoeducation comparison condition (randomly assigned at Stage 1). It also tested the addition of minimal human contact (ie, supplemental telecoaching randomly assigned at Stage 2 [78]) for CBM-I participants classified as having a higher risk of dropout early in the study. Additional details can be found in the main outcomes paper by Eberle et al [74].

After completing the anxiety screener (DASS-21-Anxiety Scale), eligible participants provided consent and were enrolled. The participants were asked to complete a baseline assessment and 1 training session per week for 5 weeks (5 sessions total, 40 scenarios each in CBM-I), with an assessment immediately after each session and a follow-up assessment 2 months after the last session. If the participants completed only part of a training or assessment task, they continued the task the next time they returned. They were compensated via e-gift cards (refer to Table 1 for details). A total of 5267 participants completed the eligibility screener, out of which 1748 (33.19%) were eligible and were enrolled. To allow a clean analysis of attrition during positive CBM-I, data [79] from the CBM-I–only intervention arm (n=699, 39.99% of enrolled participants; ie, CBM-I condition excluding participants at high risk who were randomized to receive supplemental coaching) were used in this paper.

In total, 252 Managing Anxiety participants, 326 Future Thinking participants, and 699 Calm Thinking participants were in the positive CBM-I intervention arm of these studies.

#### Definition of Attrition

In this paper, we predict attrition in multisession DMHIs. A paper by Eysenbach [18] defined two types of attrition: (1) *nonuse attrition*, which refers to participants who stopped using the intervention (ie, who did not complete the training sessions), and (2) *dropout attrition*, which refers to participants who were lost to follow-up because they stopped completing research assessments (eg, who did not complete follow-up assessment). In MindTrails studies, training and assessment tasks are intermixed and must be completed in series. For example, the participants cannot complete Session 1 assessment until they complete Session 1 training, they cannot complete Session 2 training until they complete Session 1 assessment, and so on. Due to this sequential design, nonuse and dropout attrition are conflated in our studies. As it is impossible to skip any training or assessment tasks, we simply use the term *attrition* in this paper.

### Ethical Considerations

All 3 studies were reviewed and approved by the IRB of the University of Virginia (Managing Anxiety: IRB #2703; Future Thinking: IRB #2690; and Calm Thinking: IRB #2220). After screening, the eligible participants provided informed consent for “a new internet-based program.” Data were stored in accordance with University of Virginia Information Security policies, and deidentified data were analyzed. In the Calm Thinking study, the participants were compensated with e-gift cards worth up to US $25: US $5 for each assessment at pretreatment and after Sessions 3 and 5, and US $10 for the follow-up assessment. Compensation is detailed by study in Table 1.

### Attrition Prediction Pipeline

DMHIs are often divided into multiple phases, sometimes called *modules*. In this paper, we refer to modules as *sessions* to mirror the language used by mental health specialists for in-person treatment (eg, holding sessions with a client). We proposed a pipeline that is built to handle multisession DMHI datasets with a diverse set of features. As our focus is on multisession studies, we assumed that the study contained ≥1 assessment or training session to achieve the study goals. Therefore, we required at least 1 observation from each participant for the selected features.

**Figure 1:**
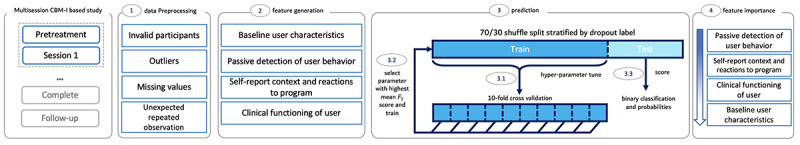
Overview of the pipeline predicting early-stage attrition in web-based, multisession cognitive bias modification for interpretation (CBM-I) interventions.

Predicting early-stage dropout in DMHIs is challenging and requires several key tasks. We first determined the prediction horizon of the selected CBM-I interventions (Aim 1). We then organized the remaining tasks into four main steps from the data science and engineering literature: (1) data preprocessing, (2) feature generation, (3) predictive modeling, and (4) feature importance. We outline these steps in the context of attrition prediction in DMHI in Figure 1 and describe each step below.

### Prediction Horizon

**Figure 2:**
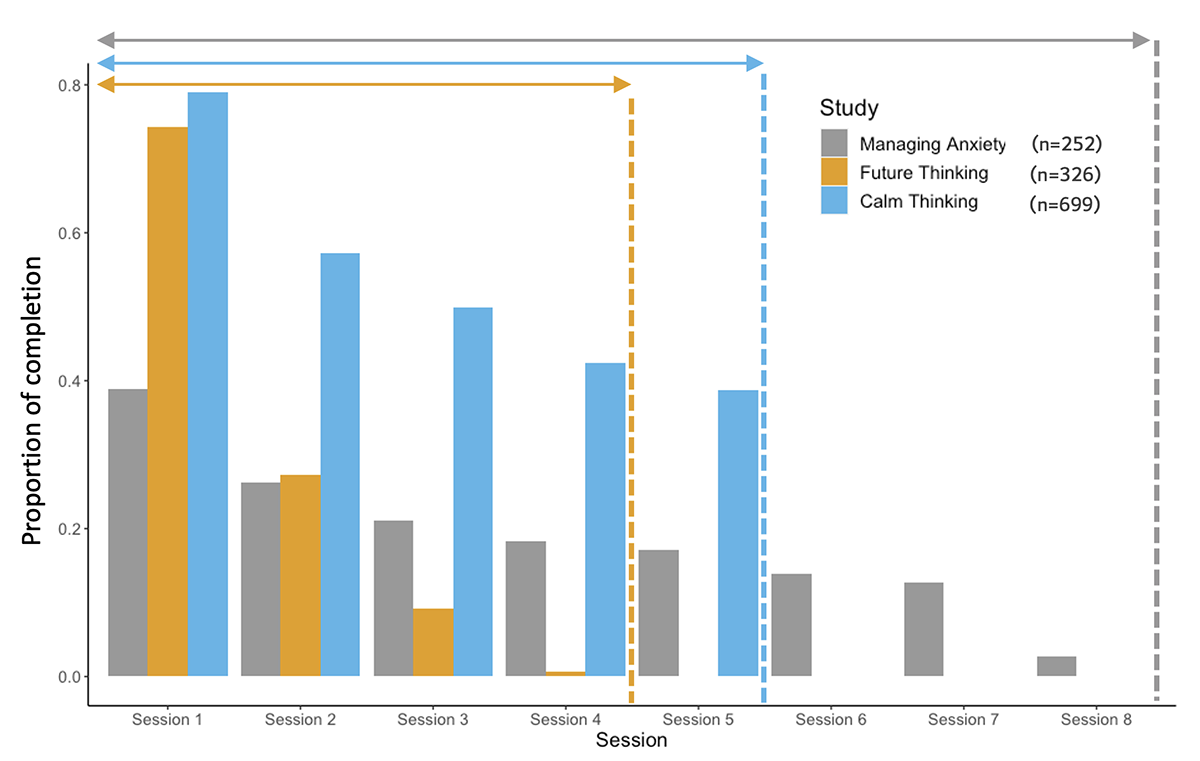
Proportion of completion per training session (out of participants who started Session 1 training) by study. The session was deemed completed if participants completed the last questionnaire in the assessment that immediately followed the training session. Dashed lines show the last training session for each study.

To analyze when users stopped using the intervention (Aim 1), the proportions of participants who completed each training session (out of the number of participants who started Session 1 training) were visualized (Figure 2). In this figure, each session was considered complete if participants completed the last questionnaire (ie, had an entry in the Task Log for the questionnaire) in the assessment that immediately followed a given training session. For the following reasons, we decided to focus on participants who had started Session 1 training and to predict which of these participants were at a high risk of dropping out before starting Session 2 training (Aim 2). First, our goal is to make inferences about user dropout during DMHIs (and not to simply use baseline assessments to predict which users will fail to even start the program). We restricted the sample to participants who had started Session 1 training because we consider these participants as part of the intent-to-treat sample. Second, the highest rate of attrition was observed between the start of the first training session and the end of the second session’s assessment, with most dropout occurring between the sessions (vs during Session 1 or Session 2). Therefore, we wanted to predict participants at a high risk of dropping out before starting Session 2 training. Notably, the identification of participants who are at a high risk of dropout early in the program might decrease the attrition rate at the end of the intervention. This is because detecting participants at high risk sooner rather than later permits targeted supports to be added to increase retention at pivotal times.

### Data Preprocessing

#### Overview

All data must be *preprocessed* before analysis, especially data collected outside a controlled laboratory environment. In the following paragraphs, we describe our methods for addressing issues such as invalid participant data, outliers, and missingness during preprocessing.

#### Invalid Participants

One of the main challenges in web-based digital mental health studies is to distinguish spam and bot-generated responses from real responses [19,79]. Malicious actors often use bots to complete questionnaires when they learn of an appealing incentive, such as monetary compensation for participating in a study. To increase the validity of the input data, we removed suspicious responses such as those that were submitted quickly (eg, <5 s for half of all questions in a given measure) or contained submissions that violated the required wait time (eg, 48 h) between sessions.

#### Outliers

To reduce the likelihood of identifying coincidental events, we first normalized the data using the *z* score metric. We then identified and removed outliers; as we did not expect to have very large or small data values [80], we excluded outliers at least 3 SDs from the mean value [81] for numerical variables. For categorical variables, we excluded outliers based on visual inspection of a frequency distribution (a histogram with the Freedman-Diaconis rule to determine the bin width).

#### Missing Values

Real-world data collection is often messy; technical issues, dropout, and loss of network connection are all common issues that arise and can lead to missing values for some or all items of a given questionnaire. In addition, participants in DMHIs are often given the option to decline to answer items when responding to a self-reported questionnaire. This may be done either implicitly (in which the question is not required) or explicitly (in which the participant is given a set of options, where one of the options is “prefer not to answer” or a similar response). The challenge associated with empty or “prefer not to answer” values is that they both function as missing values.

Missing values are a fundamental issue in digital health interventions for several reasons [82]. Most machine learning techniques are not well prepared to deal with missing data and require that the data be modified through imputation or deletion of the missing records. In addition, missing data may significantly impact the predictive analysis as well as descriptive and inferential statistics [82]. To address these issues, we used several imputation approaches to handle the challenge of missing data in some or all items in the required features and time points for different types of variables. Without imputation, these missing data could lead to more bias, decreased statistical power, and lack of generalizability.

We handled missing data for all features, for each unique time point, using the following methods: out of the initial set of features (221 for Managing Anxiety, 109 for Future Thinking, and 241 for Calm Thinking), we first removed features or variables at a given time point that have missing values in >80% of all valid participants. The percentages of features removed for this reason in Managing Anxiety, Future Thinking, and Calm Thinking studies were 33.94% (75/221), 20.18% (22/109), and 47.30% (114/241), respectively, yielding a final set of 146, 87, and 127 features, respectively. Next, we imputed categorical variables at a given time point with the most frequent answers at that time point of participants with the same demographics. To do so, we grouped participants based on 2 of the demographic characteristics (ie, education and gender, which were the most complete). To impute the numerical individual item variables at a given time point, we used the *k*-nearest neighbors method [83] to replace the missing values in the same demographic group with the mean value at that time point from the 5 nearest neighbors found in the training set. We used a Euclidean distance metric [84] to impute the missing values.

#### Unexpected Multiple Observations

Unexpected multiple observations may be present within a DMHI dataset for several reasons. Participants might complete the eligibility screener multiple times to gain access to the intervention if they were previously screened or to achieve a more desirable score. Technical issues can also cause duplicate values. For example, a brief server error may cause a questionnaire to be submitted more than once. We used one of the following two strategies to handle unexpected multiple observations: (1) calculate the average values of each item across the observations or (2) keep the latest observation. We selected one of the abovementioned strategies based on the temporal latency between unexpected multiple observations. If the temporal latency between unexpected multiple observations was less than the mean latency across all participants, we applied the first strategy. Otherwise, the second strategy was selected.

### Feature Generation

#### Baseline User Characteristics

(Note: Measures without citations in this section and the sections below were developed by the MindTrails research team.) At the baseline assessment of the 3 studies, the following demographic variables were assessed: age, gender, race, ethnicity, education, employment status, marital status, income, and country. History of mental health disorders and treatment were also assessed. In the Managing Anxiety and Calm Thinking studies, participants were also asked about the situations that make them anxious; these situations are called *anxiety triggers*. We included these measures in our baseline user characteristics feature set (Table 2).

**Table 2:** Selected features by set extracted from cognitive bias modification for interpretation studies.

Set and task (from Task Log^a^)		Description	Study		Session
**Baseline user characteristics**					
	Demographics	Assesses age, gender, race, ethnicity, education, employment status, marital status, income, and country	MA^b^, FT^c^, and CT^d^	Baseline	
	Mental health history	Assesses mental health disorders and treatments	MA, FT, and CT	Baseline	
	Anxiety triggers	Assesses situations that prompt anxiety	MA and CT	Baseline	
**Self-reported context and reactions to program**					
	Credibility	Assesses importance of reducing anxiety or changing thinking (Importance Ruler) and confidence in intervention [85]	MA, FT, and CT	Baseline	
	Return intention	Assesses days until returning	MA, FT, and CT	Session 1	
	Affect	Assesses state anxiety (Subjective Units of Distress; in MA and CT) or current positive and negative feelings (in FT)	MA, FT, and CT	Session 1	
	Impact of anxious imagery prime	Assesses peak anxiety during imagery prime	MA and CT	Session 1	
	Session review	Assesses location, level of distraction, and ease of use of program	CT	Session 1	
**Passive detection of user behavior**					
	All assessment and training tasks	Computed time on a page, time of the day, and day of the week	MA, FT, and CT	Baseline and Session 1	
	All assessment and training tasks	Computed cumulative time elapsed to complete all components of a given task and latency between completing one task and starting the next	CT	Baseline and Session 1	
	Training task (for FT) and all assessment and training tasks (for CT)	Device (from Training table for FT, from Task Log for CT)	FT and CT	Baseline and Session 1	
**User clinical functioning**					
	Interpretation bias (Recognition Ratings)	Assesses positive and negative interpretations of ambiguous situations (each valence scored separately, including both threat-related and threat-unrelated items^e^)	MA and CT	Baseline	
	Interpretation bias (Brief Body Sensations Interpretation Questionnaire)	Assesses positive and negative interpretations of ambiguous situations (each valence scored separately, including items for both internal and external events and excluding neutral items)	MA and CT	Baseline	
	Expectancy bias	Assesses positive and negative expectations for ambiguous future situations (Expectancy Bias Task; each valence scored separately)	FT	Baseline and Session 1	
	Anxiety (OASIS^f^)	Assesses anxiety symptoms (OASIS)	MA and CT	Baseline and Session 1	
	Anxiety (DASS21-AS^g^)	Assesses anxiety symptoms	MA and CT	Baseline	
	Anxiety and depression (PHQ-4^h^)	Assesses anxiety (Generalized Anxiety Disorder-2 scale) and depression (2-item PHQ) symptoms (each measure scored separately)	FT	Baseline	
	Depression (DASS21-DS^i^)	Assesses depression symptoms	MA	Baseline	
	Daily drinking	Assesses alcohol use (Daily Drinking Questionnaire)	MA	Baseline	
	Anxiety identity	Assesses centrality of anxiety to identity (Anxiety and Identity Circles)	CT	Baseline	
	Mechanisms	Assesses cognitive flexibility (Cognitive Flexibility Inventory), experiential avoidance (Comprehensive Assessment of Acceptance and Commitment Therapy Processes), cognitive reappraisal (Emotion Regulation Questionnaire), and intolerance of uncertainty (Intolerance of Uncertainty Scale-12; each measure scored separately)	CT	Baseline	
	Wellness (What I Believe)	Assesses self-efficacy (NGSES^j^), growth mindset (PBS^k^), and optimism (LOT-R^l^; each measure scored separately)	FT	Baseline	
	Wellness	Assesses self-efficacy (NGSES), growth mindset (PBS), optimism (LOT-R), and life satisfaction ([86]; each measure scored separately)	CT	Baseline	
	Wellness (QOL^m^)	Assesses life satisfaction	MA	Baseline	

^a^Task Log is a log table that tracks the completion of each assessment and training task for each participant in a given study; when the task’s content is not evident in the task’s name, the content is listed and the name is in parentheses.

^b^MA: Managing Anxiety.

^c^FT: Future Thinking.

^d^CT: Calm Thinking.

^e^Positive and negative interpretation bias assessed using Recognition Ratings are typically scored using only the threat-related items, but given that these are only 2 features, we do not expect this to markedly impact the algorithm.

^f^OASIS: Overall Anxiety Severity and Impairment Scale.

^g^DASS-21-AS: 21-item Depression Anxiety Stress Scales-Anxiety Scale.

^h^PHQ-4: 4-item Patient Health Questionnaire.

^i^DASS-21-DS: 21-item Depression Anxiety Stress Scales-Depression Scale.

^j^NGSES: New General Self-Efficacy Scale.

^k^PBS: Personal Beliefs Survey.

^l^LOT-R: Life Orientation Test-Revised.

^m^QOL: Quality of Life Scale.

#### Self-Reported User Context and Reactions to Program

The importance of reducing anxiety or changing thinking (Importance Ruler, modified from Case Western Reserve University [63]) and confidence in the intervention (modified from Borkovec and Nau [85]) were assessed at the baseline assessment of every study. In addition, after completing a given session’s assessment, participants were asked for the date they would return for the next session. State anxiety (in Managing Anxiety and Calm Thinking; Subjective Units of Distress, SUDS, modified from Wolpe [87]) or current positive and negative feelings (in Future Thinking) were assessed before and after participants completed each session’s training. The Managing Anxiety and Calm Thinking studies also assessed participants’ peak anxiety when imagining an anxiety-provoking situation in their lives as part of the anxious imagery prime completed toward the start of training. At the end of each session in the Calm Thinking study, the participant’s location, level of distraction, and ease of use of the program were assessed. All of these measures were included in the self-reported user context and reactions to the program feature set (see details in Table 2).

#### Passive Detection of User Behavior

To further understand participants’ context and behavior when interacting with the platform, the following variables were calculated: time spent on a page, time of day, day of the week, and latency of completing assessments. The type of device (ie, desktop, tablet, smartphone) was also included as a feature given that multiple devices could be used to access the program, each with different characteristics (eg, screen size, input methods, and mobility) that could influence user behavior. In most cases, these variables were extracted for each assessment and training task for each session. For details about which features were extracted for which studies, see Table 2.

#### User Clinical Functioning

Primary and secondary outcome measures used to evaluate the effectiveness of the intervention were included in the clinical functioning feature set. These measures assessed interpretation bias (Recognition Ratings, RR, modified from Matthews and Mackintosh [65]; and Brief Body Sensations Interpretation Questionnaire, BBSIQ, modified from Clark et al [88]), expectancy bias (Expectancy Bias Task, modified from Namaky et al [77]), anxiety symptoms (Overall Anxiety Severity and Impairment Scale, OASIS, adapted from Norman et al [89]; DASS-21-Anxiety Scale; and Generalized Anxiety Disorder-2 scale, GAD-2, modified from Kroenke et al [90]), comorbid depression symptoms (DASS-21-Depression Scale; and Patient Health Questionnaire-2, PHQ-2, modified from Kroenke et al [61]), and alcohol use (Daily Drinking Questionnaire, DDQ [91]). They also assessed the centrality of anxiety to identity (Anxiety and Identity Circles, modified from Ersner-Hershfield et al [92]) and other cognitive mechanisms, including cognitive flexibility (Cognitive Flexibility Inventory, CFI, adapted from Dennis and Vander Wal [93]), experiential avoidance (Comprehensive Assessment of Acceptance and Commitment Therapy Processes, CompACT, modified from Francis et al [94]), cognitive reappraisal (Emotion Regulation Questionnaire, ERQ, modified from Gross and John [95]), and intolerance of uncertainty (Intolerance of Uncertainty Scale-Short Form, IUS-12, modified from Carleton et al [96]). Finally, they assessed self-efficacy (New General Self-Efficacy Scale, NGSES, modified from Chen et al [97]), growth mindset (Personal Beliefs Survey, PBS, modified from Dweck [98]), optimism (Life Orientation Test-Revised, LOT-R, modified from Scheier et al [99]), and life satisfaction ([86]; Quality of Life Scale, QOL [100]). For details about which features were extracted for which studies, see Table 2.

### Predictive Modeling

#### Overview

For each study, predictors of attrition were investigated after participants started Session 1 training, imputing any missing values for features collected during Session 1 training or assessment.

To identify participants at high risk of dropping out before starting the second training session, the following predictors of attrition were investigated: baseline user characteristics (at the pretest assessment), self-reported user context and reactions to the program, passively detected user behavior, and clinical functioning of users. We used data from the pretest, the first training session, and the assessment following the first training session.

#### Dropout Label

For each participant, we calculated a binary ground truth label for their actual dropout status before starting the second training session, where 0 indicates the participant started training for the second session and 1 indicates the participant did not start training for the second session (ie, dropped out). A participant was deemed as having started a given session’s training if they had an entry in the Task Log for the Affect task, which was administered immediately before the first page of training materials for each session.

#### Class Imbalance

Class imbalance is a common problem for supervised learning tasks such as attrition prediction. Such datasets have 1 or more classes (eg, “did not dropout” in the case of Calm Thinking) that have a greater number of observations than other classes (eg, “dropped out” in Calm Thinking). Class imbalance can worsen the performance of machine learning models by biasing them toward learning the more commonly occurring classes. We used the synthetic minority oversampling technique [101] to help rectify the class imbalance.

The synthetic minority oversampling technique resolves this challenge by generating synthetic samples for the minority class, with the aim of balancing the distribution of samples between the 2 classes. The technique operates by selecting 2 or more samples from the minority class and computing the difference between their features. This difference is then added to the feature values of one of the selected samples to create a new synthetic sample. This process is repeated to generate a sufficient number of synthetic samples, which are then added to the original dataset to achieve an optimal balance between the majority and minority classes. It has proven to be very effective in dealing with class imbalance problems for tabular datasets [102] (Figure 2).

#### Classification

Binary classification is a well-studied problem in the machine learning literature [103,104], and a plethora of models and approaches exist for predicting attrition. We selected leading machine learning models, beginning with simpler, more interpretable models and progressing to more expressive models for identifying the best predictors of early-stage dropout. We trained and validated a range of models, described in detail below and listed in Table 3. Models that learn a linear decision boundary are typically the first approach for binary classification problems. These models separate participants into 2 classes defined by the estimated decision boundary, in our case participants who drop out and those who remain. The logistic regression model estimates this decision boundary by minimizing the mean squared error of predictions in the training set [105]. Similarly, the support vector machine (SVM) estimates this boundary by maximizing the distance from the edge of each class. Some nonlinearity is also introduced into the SVM by projecting its feature space with the radial basis function (RBF) kernel [106].

Other models estimate a nonlinear decision boundary. A decision tree model estimates a continuous piecewise boundary, with each piece indicating a different set of conditions that leads to a particular leaf node of the tree [107]. We further evaluated several tree-based ensemble models. In ensemble models, multiple submodels are composed to form a prediction. The random forest model uses decision trees as its submodel, creating a “forest” (set) of such trees. The random forest estimates the best feature subset to give to each tree while maximizing the average prediction accuracy over all trees [107]. Similarly, AdaBoost comprises multiple shallow decision trees, giving a weighting to each tree according to the overall prediction accuracy [107].

Finally, gradient boosting algorithms (and the related extreme gradient boosting [XGBoost] method [108]) were used to train ensembles of decision trees. Gradient boosting minimizes an objective function that is differentiable with respect to all submodel parameters, and the submodel parameters are adjusted via gradient descent. XGBoost [108] is based on the same concept, but also includes parameter regularization to prevent overfitting and second-order derivatives to control gradient descent. The regularized greedy forest (RGF) model was also evaluated. RGF not only includes tree-structured regularization learning, but also employs a fully corrective regularized greedy algorithm [109]. Finally, a multilayer perceptron model was used. This neural network model implements a feed-forward architecture that backpropagates error with stochastic gradient descent [110].

We employed 10-fold cross-validation stratified by dropout label (ie, dropout vs not dropout) across 100 iterations. Hyperparameter tuning was performed using group 5-fold cross-validation on the training set. Hyperopt [111] was used to optimize hyperparameters including the number of estimators, learning rate, maximum tree depths, *C* parameter, and *γ*. We evaluated up to 30 combinations of these parameters to maximize the model’s average macro–*F*_1_-score across 5 folds. The set of hyperparameters that achieved the highest average macro–*F*_1_-score across the 5 folds was chosen to train the model on the entire training set during the outer split.

**Table 3:** Performance of attrition prediction models within a given study based on macro–F1-scores, area under curve, and accuracy scores. The models were trained on the Managing Anxiety (MA) [19], Future Thinking (FT) [44], and Calm Thinking (CT) [74,79] studies and were tested on their respective test sets.

Data and model		Evaluation metric^a^		
		Macro*–F*_1_-score↑^b^	Area under the curve↑	Accuracy↑
**Training and test data: Managing Anxiety with 146 features**				
	Logistic regression	.698	.774	.717
	Support vector machine	.723	.802	.760
	Decision tree	.555	.610	.644
	Random forest	.819	.827	.843
	Gradient boosting	.802	.808	.808
	Extreme gradient boosting	*.832* ^c^	.848	*.858*
	Regularized greedy forest	.794	*.853*	.823
	Multilayer perceptron	.690	.772	.723
**Training and test data: Future Thinking with 87 features**				
	Logistic regression	.682	.752	.689
	Support vector machine	.719	.787	.728
	Decision tree	.688	.745	.693
	Random forest	.767	.840	.768
	Gradient boosting	.758	.823	.759
	Extreme gradient boosting	*.770*	*.844*	*.771*
	Regularized greedy forest	.728	.817	.735
	Multilayer perceptron	.694	.778	.703
**Training and test data: Calm Thinking with 127 features**				
	Logistic regression	.878	.874	.878
	Support vector machine	.869	.861	.869
	Decision tree	.786	.895	.788
	Random forest	.914	.917	.910
	Gradient boosting	.901	.908	.901
	Extreme gradient boosting	*.917*	*.926*	*.918*
	Regularized greedy forest	.902	.908	*.918*
	Multilayer perceptron	.878	.879	.878

^a^Each metric can range from 0 to 1, with macro–*F*_1_-score and area under curve values >.5 and accuracy values >.7 generally considered reasonable; refer to the *Evaluation Metrics* section for details.

^b^↑ indicates that higher values are more desirable for a given metric.

^c^The highest values for each metric are italicized.

#### Model Optimization

To enhance model performance and efficiency, optimization techniques were applied. For instance, in the SVM model, we selected the RBF kernel with *γ* determined as 1/(number of features × *X.var ()*) to control the influence of training examples. In decision tree models, all features were considered for finding the best splits, while feature subsampling was employed to reduce model correlation and variance.

Our selected criterion for the decision model is entropy, which measures the degree of disorder of the features in relation to the target. The optimum split is chosen by the feature with the lowest entropy. It gets its maximum value when the probability of the classes is the same. A node is pure when the entropy has its minimum value, which is zero. For the random forest model, we take all the features that make sense in every tree.

In the XGBoost model, we set the subsample ratio of columns for each level equal to 0.4. Sampling occurs once for every new tree. The *γ* parameter in XGBoost is used as a threshold for creating new splits in the tree; it represents the minimum loss reduction required to make a further partition on a leaf node of the tree. We set *γ*=8. To control the balance of positive and negative weights in a binary classification problem, we set the parameter *scale_pos_weight* = *sum*(negative instances) / *sum*(positive instances). This parameter allows adjustment of the relative weight of positive instances in the cost function, by setting it to the ratio of negative to positive instances. This can help to handle imbalanced datasets where one class is underrepresented, as in our case. The *eta* parameter, learning rate, controls the step size shrinkage used in updating the weights to prevent overfitting. We tuned *eta* for our models and dataset and got the value 0.01. After each boosting step in XGBoost, we can directly get the weights of newly added features, and *eta* shrinks the feature weights and the weights of all the features in the model to make the boosting process more conservative. The *α*=*.*3 parameter in XGBoost is used as a regularization term on the weights; it represents the L1 regularization term, which is used to add a penalty term to the cost function that is proportional to the absolute value of the weights. This helps to prevent overfitting by shrinking the weights toward zero. The *λ*=0*.*4 parameter in XGBoost is also used as a regularization term on the weights; it represents the L2 regularization term, which is used to add a penalty term to the cost function that is proportional to the square of the weights. This helps to prevent overfitting by shrinking the weights toward zero.

For RGF, we used the min-penalty regularization with sum-to-zero sibling constraints to improve the interpretability of the model. For logistic regression, we set the regularization to *elasticnet* and the regularization strength to 1, *C*=1. For a multilayer perceptron, the activation function is set to the rectified linear unit function, represented as *f*(*x*)=*max*(0,*x*). The initial learning rate for the Adam algorithm is also set to 0.001. It is worth noting that we kept the other hyperparameters of the models at their default values to avoid overfitting and to ensure the stability of the models.

#### Evaluation Metrics

We used 3 standard metrics to evaluate attrition prediction: macro–*F*_1_-score, area under the curve (AUC; ie, area under the receiver operating characteristic [ROC] curve), and accuracy. For macro–*F*_1_-score, an *F*_1_-score is first computed for each class. The *F*_1_-score is the harmonic mean of *precision* (proportion of positive predictions that are correct) and *recall* (proportion of positive classes that are correctly predicted; *true positive rate*), and it rewards true positives and penalizes false positives and false negatives. *F*_1_-scores range from 0 (when no positive predictions are correct) to 1 (when all positive predictions are correct, and no incorrect negative predictions are made). Macro–*F*_1_-score is the arithmetic mean of *F*_1_-scores across classes and is widely used when classes are imbalanced because it avoids bias toward the majority class by weighting each class’s *F*_1_-score equally.

AUC, a widely adopted performance metric, measures the trade-off between the true positive rate and the *false positive rate* (proportion of negative classes that are incorrectly predicted as positive) by plotting these rates against one another for various classification thresholds (ie, probabilities above which a positive prediction is made) and quantifying the area under the resulting ROC curve; this area provides an aggregate measure of performance across all possible thresholds. AUC ranges from 0 (when no positive classes are correctly predicted and all negative classes are incorrectly predicted) to 1 (when all positive classes are correctly predicted and no negative classes are incorrectly predicted), indicating the model’s ability to differentiate between positive and negative classes (a value of .5 reflects random prediction).

Accuracy, in turn, is the proportion of all predictions (positive and negative) that are correct and ranges from 0 (no predictions are correct) to 1 (all predictions are correct), providing a straightforward assessment of the model’s overall performance, although it can be misleading in isolation when classes are imbalanced. For macro–*F*_1_-score and AUC, values above .5 are generally considered to reflect reasonable performance, while for accuracy, a value above .7 is considered reasonable.

### Feature Importance

Aim 3 of this paper is to explore which feature sets are most important for the identification of participants at high risk. To analyze this, the effect of each feature set on the prediction models was calculated (ie, Gini importance [112]). We report the mean Gini importance score across 2 iterations. Gini importance scores reflect the importance of a feature set relative to others (not absolute importance) and can range from 0 to 1, with higher scores reflecting greater importance.

## Results

### Model Performance

The results demonstrate that with these predictors (number of features for the Managing Anxiety, Future Thinking, and Calm Thinking studies: 146, 86, and 127, respectively), we were able to identify participants with a high risk of dropping out before starting the second training session of each study (macro–*F*_1_-score for XGBoost in the Managing Anxiety, Future Thinking, and Calm Thinking studies: .832, .770, and .917, respectively; Table 3). These results show the effectiveness of different feature sets in predicting attrition in the early stages of the DMHIs. Moreover, these results show the superiority of the XGBoost and the random forest models in predicting attrition (see Table 3). XGBoost always places more importance on functional space when reducing the cost of a model, while random forest tries to place more importance on hyperparameters to optimize the model.

### Sensitivity to Imputation

To assess the impact of imputation on our prediction models, we conducted an ablation experiment (ie, systematic removal of a component of the model to test its effect) that eliminated the imputation step from our pipeline. We used the XGBoost classification model in this experiment, as it demonstrated superior performance throughout our analyses. The results, presented in Table 4, reveal a substantial decrease in performance when imputation using *k*-nearest neighbors is removed from the pipeline, highlighting the importance of imputation in our prediction models.

**Table 4:** Sensitivity of attrition prediction model performance to imputation^a^.

Data and extreme gradient boosting model version		Evaluation metric		
		Marco–*F*_1_-score↑^b^	Area under the curve↑	Accuracy↑
**Managing Anxiety with 146 features**				
	No imputation	.715	.801	.716
	Imputation	*.832* ^c^	*.848*	*.858*
**Future Thinking with 87 features**				
	No imputation	.726	.796	.729
	Imputation	*.770*	*.844*	*.771*
**Calm Thinking with 127 features**				
	No imputation	.905	.904	.910
	Imputation	*.917*	*.926*	*.918*

^a^Ablated versions of the proposed pipeline without imputing missing values are compared with the full pipeline in terms of macro–*F*_1_-score, area under the curve, and accuracy scores. All models used extreme gradient boosting and were trained and tested on all feature sets of the Managing Anxiety, Future Thinking, and Calm Thinking studies.

^b^↑ indicates that higher values are more desirable for a given metric (which each can range from 0 to 1, with macro–*F*_1_-score and area under curve values >.5 and accuracy values >.7 generally considered reasonable; refer to the *Evaluation Metrics* section for details).

^c^The highest values for each metric are italicized.

### Feature Importance

**Figure 3:**
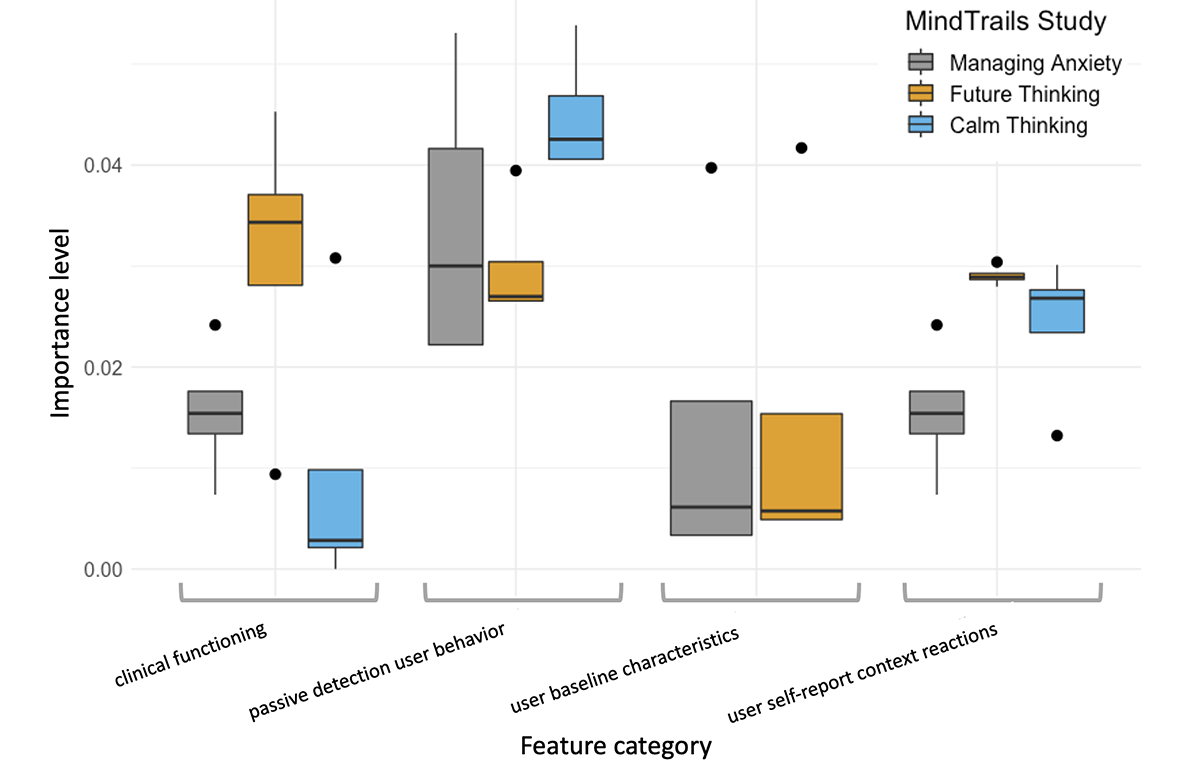
Importance level of each feature set relative to other feature sets for early attrition prediction in cognitive bias modification for interpretation studies. Gini importance scores averaged across 2 iterations are shown. We used the XGBoost classifier because it performed the best. These scores reflect the importance of a feature set relative to others (not absolute importance) and can range from 0 to 1, with higher scores reflecting greater importance. Horizontal bars reflect the median score; dots represent outliers, which are observations that fall outside of the box plot; and whiskers represent the minimum and maximum observations within 1.5 times the IQR from the lower and upper quartiles, respectively. No important baseline user characteristic features emerged for the Calm Thinking study. XGBoost: extreme gradient boosting.

To investigate how the different feature sets affect the performance of attrition prediction, we calculated the average importance score (ie, weight) for the important features from the selected high-performing classifier after 100 iterations. Overall, a few trends emerged in identifying individuals at high risk of dropout: the passively detected user behavior feature set, and then the self-reported user context and reaction to the program feature set, are consistently more important than the user baseline characteristics and user clinical functioning feature sets for predicting early-stage attrition in a multisession CBM-I intervention (Figure 3). More specifically, we found that features involving passive detection of user behavior, such as time spent on a web page of CBM-I training exercises, time of day, latency in completing assessments, and the type of device used, were the most informative predictors of attrition, with mean Gini importance scores across 2 iterations of .033 (95% CI .019-.047), .029 (95% CI .023-.035), and .045 (95% CI .039-.051) for the Managing Anxiety, Future Thinking, and Calm Thinking studies, respectively (Figure 3). However, it should be noted that these observed patterns were not statistically tested for significance.

## Discussion

### Principal Findings

In this research, we investigated the potential of predicting early attrition from 3 studies of multisession, web-based, positive CBM-I training programs using a combination of features derived from training and assessment data, including baseline user characteristics, self-reported user context and reactions to the program, passive detection of user behavior, and user clinical functioning. Our proposed pipeline was able to identify participants who were at a high risk of dropping out early in these studies. Our pipeline provides a framework (ie, data preprocessing, feature generation, predictive modeling, and feature importance) for predicting attrition in DMHIs broadly, although the particulars (eg, features) will vary with each application. Our results also show that passive features describing user behavior when interacting with a DMHI can be a valuable feature for identifying individuals at high risk of dropping out. In our analyses, interestingly, passive features of user behavior were more informative to this prediction than other features, including user clinical functioning, emphasizing the utility of considering users’ real-time behavior in predicting early attrition.

While these findings need to be validated in future studies, they highlight the value of considering the collection and use of such features in algorithms for predicting attrition in future DMHI designs. Key next steps include the need to make these data-driven approaches transferable to real-world care settings (ie, beyond research settings). Clinicians tend not to integrate DMHIs into their clinical practice, in part due to lack of training and understanding about how DMHIs work, which DMHIs to choose, and how to integrate them [28]. Helping clinicians determine which of their patients is likely to stick with a DMHI (and benefit from it) may help address some of these clinician concerns, and further personalization of the approaches may be useful. Along these lines, more longitudinal features capturing user interaction with DMHIs could enable a level of personalization and customization that has historically been challenging to achieve with only baseline characteristics. It will also be important to address the challenges raised by the complexities of interpreting these algorithms (ie, determining which factors were key to predicting attrition). When the algorithms seem impenetrable, it may increase clinicians’ discomfort with applying them in their practice.

The findings also highlight the value of using *both* passive user behavioral data collected during the DMHI and the users’ self-report data. Predicting clinical outcomes from single indicators has routinely not been successful. Speaking to the historical challenges in predicting response to depression treatments, van Bronswijk et al [113] noted that “no single moderator is likely to be robust enough, on its own, to reliably guide treatment selection..., and indeed none have been identified.” This has led many researchers to recognize the value of novel methods, such as machine learning, that allow for multivariate prediction. This paper extends this approach further by integrating multiple sources of information, beyond only self-report features. This has several advantages, including reducing user burden by not relying solely on self-reported measures; it allows for prediction to be carried out based on meaningful data about users that they may not have introspective access, or comfort, to report effectively.

### Model Performance and Feature Importance

Features extracted from the early stages of a given study (ie, baseline assessment and Session 1 training or assessment; Table 2) were highly predictive of attrition before starting Session 2 training (Table 3). Particularly important was the feature set involving passive detection of user behavior (Figure 3), which consisted of time spent on page, time of the day, day of the week, time spent on tasks, latency between tasks, and device type. Although it is unclear which passive features were most informative (a useful future direction), it may be that certain passive features (eg, time on a web page) contain real-time information about engagement, motivation, or ability to use the program not captured by other measures (eg, self-reports of the importance of reducing anxiety or confidence in the program at baseline or self-reports of ease of using the program at the end of Session 1). However, the feature importance level varied by classifier and study, highlighting the complexity of identifying individual predictors of attrition. Nevertheless, future studies may benefit from including similar feature sets, especially behavioral features.

Furthermore, our analyses revealed that predicting attrition in DMHI studies is not an easy problem; otherwise, simpler models such as the logistic regression and SVM models may have provided sufficient predictive power. The more complex models that leverage ensembles (random forest, gradient boosting, XGBoost, etc) performed substantially better without overfitting to the data by making use of cross-validation and parameter tuning. These models are also inherently interpretable, making it easier to explain results to various audiences, including clinicians and other stakeholders. Overall, these results suggest that ensemble and forest models may provide a strong baseline when predicting attrition in CBM-I studies.

### Transfer of Knowledge

Given the sparsity of the original dataset, we expected that models would perform better when given informative priors from similar studies. For example, we can use data from the Managing Anxiety study to provide informative priors to the prediction model that is then trained to predict attrition in the Calm Thinking study. We found that, despite their common use of the MindTrails web infrastructure and use of CBM-I interventions, the 3 studies (Managing Anxiety, Future Thinking, and Calm Thinking) had substantially different data distributions (ie, attrition rate and raw values for given features). The studies also had different model performance, not only when each study used all of its own features (Table 3), but also when the studies used only the features they shared (Table 5). Thus, although our findings provide insights into next steps for this research, their generalizability to other CBM-I studies and DMHIs more broadly is somewhat limited.

This wide variation in data distributions and model performance points to the larger challenge of generalizability in eHealth studies. To address this issue in future work on eHealth attrition prediction using machine learning, we recommend researchers to (1) consider what aspects of our proposed pipeline may be relevant to their specific context and (2) incorporate more advanced transfer learning techniques. Transfer learning is a machine learning method that leverages knowledge learned from one problem and applies it to a related but different problem. Advanced transfer learning techniques can enhance DMHIs by using existing knowledge, addressing class imbalance and feature extraction, and incorporating insights from large datasets to drive actionable solutions for reducing attrition and increasing engagement in DMHIs.

**Table 5:** Evaluation of transfer of knowledge between studies based on macro–F1-score, area under the curve, and accuracy scores. The models were trained on the Managing Anxiety (MA), Future Thinking (FT), and Calm Thinking (CT) studies and were tested on the other studies’ test sets.

Data and model				Evaluation metric		
			Macro–*F*_1_-score↑^a^		Area under the curve↑	Accuracy↑
**Training data: MA**						
	**Test data: CT sharing 44 features with MA**					
		Logistic regression	.470		.504	.490
		Support vector machine	.445		.517	.614
		Decision tree	.473		.510	.550
		Random forest	*.496* ^b^		.518	.554
		Gradient boosting	.413		.487	.593
		Extreme gradient boosting	.462		*.596*	*.646*
		Regularized greedy forest	.470		.493	.540
		Multilayer perceptron	.491		.493	.540
	**Test data: FT sharing 32 features with MA**					
		Logistic regression	*.519*		.549	.538
		Support vector machine	.444		.538	*.618*
		Decision tree	.469		.544	.594
		Random forest	.515		.538	.554
		Gradient boosting	.439		.532	.613
		Extreme gradient boosting	.379		.490	.611
		Regularized greedy forest	.502		.535	.560
		Multilayer perceptron	.516		*.566*	.569
**Training data: FT**						
	**Test data: CT sharing 30 features with FT**					
		Logistic regression	.527		.562	.535
		Support vector machine	.545		.623	.598
		Decision tree	.549		.583	.573
		Random forest	*.582*		*.639*	.585
		Gradient boosting	.536		.633	*.604*
		Extreme gradient boosting	.447		.635	.571
		Regularized greedy forest	.557		.608	.580
		Multilayer perceptron	.564		.595	.581
	**Test data: MA sharing 32 features with FT**					
		Logistic regression	.521		.553	.529
		Support vector machine	.495		.589	*.586*
		Decision tree	.513		.556	.550
		Random forest	*.572*		*.612*	.575
		Gradient boosting	.469		.586	.574
		Extreme gradient boosting	.370		.584	.571
		Regularized greedy forest	.535		.565	.566
		Multilayer perceptron	.541		.585	.560
**Training data: CT**						
	**Test data: MA sharing 44 features with CT**					
		Logistic regression	.811		.816	.812
		Support vector machine	.820		.866	.820
		Decision tree	.766		.717	.767
		Random forest	.835		.828	.835
		Gradient boosting	.843		.856	*.853*
		Extreme gradient boosting	*.853*		.857	*.853*
		Regularized greedy forest	*.853*		.858	*.853*
		Multilayer perceptron	.821		*.878*	.821
	**Test data: FT sharing 30 features with CT**					
		Logistic regression	.615		.668	.617
		Support vector machine	.789		.775	.790
		Decision tree	.738		.805	.740
		Random forest	*.853*		*.885*	*.853*
		Gradient boosting	.831		.843	.831
		Extreme gradient boosting	.832		.841	.832
		Regularized greedy forest	.832		.841	.832
		Multilayer perceptron	.743		.719	.744

^a^↑ indicates that higher values are more desirable for a given metric (each can range from 0 to 1, with macro–*F*_1_-score and area under the curve values above .5 and accuracy values above .7 generally considered reasonable; see the *Evaluation Metrics* section for details).

^b^The highest values for each metric are italicized.

### Applied Example

Low engagement in a DMHI may manifest as low initial uptake, substantial early dropout, or failure to adhere long term to the intervention techniques intended to change behavior. Predicting attrition is complicated by the many reasons for which a person may drop out (eg, the program is not meeting their needs or has already met their needs). Still, identifying participants at a high risk for dropout at an early stage may enable allocation of further support specifically to users who may need it, thus improving engagement while retaining scalability [31]. For example, we implemented a probability prediction algorithm in the Calm Thinking study (instead of the binary classification algorithm used in this paper) to predict each participant’s probability of not completing the second session. This probability, the user’s *attrition risk score*, was then compared with a threshold *τ* set by the project coordinator (based on a goal to have roughly equal cell sizes after the second randomization point in the study’s sequential, multiple assignment, randomized trial design). Participants (n=547) whose attrition risk score was ≥*τ* were deemed to have a higher risk of dropping out and were then randomized to receive supplemental telecoaching (n=282) or not (n=265). Those in the coaching condition received an email connecting them with their coach, who proposed a phone call to discuss study goals, reinforce use, and address any technical issues or other study questions. (We excluded higher-risk participants randomized to supplemental telecoaching [n=282] from analyses for this paper.) For more details about this implementation, refer to the main outcomes paper on the Calm Thinking study by Eberle et al [74].

### Limitations

One limitation of our analyses is that we focused on participants who started Session 1 training and excluded many participants who dropped out before that point. Another limitation is that we had to use the existing features of the studies, which narrowed our options for feature extraction. It is possible that the model would be further improved with more detailed features (eg, user continuous location [GPS]; passive detection of more finely grained user behavior at the level of individual items vs at the level of scale scores or the entire training or assessment task). In addition, the feature importance results should be interpreted cautiously; readers should refrain from inferring a causal relationship between these features and early attrition. Further research is needed to establish the extent to which such features cause or are a consequence of risk for attrition; it might also be informative to evaluate different imputation and modeling strategies. Furthermore, we used imputation strategies for all missing numeric values, even in cases where dropout meant the meaning of a given measure no longer applied (eg, for Return Intention, imputing number of days expected to return for Session 2 even when the participant did not complete Session 1; for Impact of Anxious Imagery Prime, imputing peak anxiety during the prime even when the participant started training but never completed the prime). Future studies should consider (1) removing features containing missing values that cannot be meaningfully imputed or (2) restricting the sample to participants who completed all features that cannot be meaningfully imputed. Finally, future work should seek to identify and, if needed, mitigate potential algorithmic biases. For example, the studies in this paper required participants to have internet access and were optimized for computer delivery, which may lead to underrepresentation in the training data for demographic groups that lack internet access or are dependent on smartphones [13,114]. While some studies have shown that including demographic features (eg, gender and race) in early dropout prediction has minimal impact on algorithmic fairness [115], it is prudent to perform a sensitivity analysis excluding these features, to compare model performance by demographic group, and to use bias-aware model calibration techniques when possible [116].

### Conclusions

This paper aimed to identify participants at a high risk of dropout during the early stage of 3 multisession, web-based CBM-I studies using a combination of self-reported and passively detected measures. Our findings suggest that features involving passive detection of user behavior, such as time spent on a web page of CBM-I training exercises, time of the day, latency in completing assessments, and the type of device used, were the most informative predictors of attrition. In addition, our results showed that using all features extracted from a given study led to the best predictive performance, highlighting the importance of using a combination of feature types when predicting attrition. Consequently, using passive indicators of user behavior, in conjunction with self-reported measures, can increase the accuracy of predicting dropout in web-based CBM-I studies. Although our pipeline provides a framework to consider while predicting attrition in DMHIs, many interesting, open questions remain about how extensively our findings generalize to other CBM-I studies (eg, in populations with diagnosed anxiety [vs trait anxiety], in mobile app–based [vs web-based] CBM-I studies, in CBM-Is embedded in managed care settings [vs on a public website]) and to DMHIs more broadly (eg, unguided web-based cognitive behavioral therapy [117]). Our analyses highlight the challenge of generalizability in DMHI studies and the need for more personalized attrition prevention strategies. Overall, our results emphasize the potential value of understanding user behavior in early stages of the program and using it as a predictor of dropout, which may guide development of more effective and efficient DMHIs.

## Abbreviations

AUC: area under the curve
BBSIQ: Brief Body Sensations Interpretation Questionnaire
CBM-I: cognitive bias modification for interpretation
CFI: Cognitive Flexibility Inventory
CompACT: Comprehensive Assessment of Acceptance and Commitment Therapy Processes
DASS-21: 21-item Depression Anxiety Stress Scales
DDQ: Daily Drinking Questionnaire
DMHI: digital mental health intervention
ERQ: Emotion Regulation Questionnaire
GAD-2: Generalized Anxiety Disorder-2 scale
IRB: Institutional Review Board
IUS-12: Intolerance of Uncertainty Scale-Short Form
LOT-R: Life Orientation Test-Revised
NGSES: New General Self-Efficacy Scale
OASIS: Overall Anxiety Severity and Impairment Scale
PHQ-2: Patient Health Questionnaire-2
QOL: Quality of Life Scale
RBF: radial basis function
RGF: regularized greedy forest
ROC: receiver operating characteristic
RR: Recognition Ratings
SUDS: Subjective Units of Distress
SVM: support vector machine
XGBoost: extreme gradient boosting

## References

[1] Schure MB, Lindow JC, Greist JH, Nakonezny PA, Bailey SJ, Bryan WL, Byerly MJ (2019). Use of a fully automated internet-based cognitive behavior therapy intervention in a community population of adults with depression symptoms: randomized controlled trial. J Med Internet Res.

[2] Stiles-Shields C, Montague E, Lattie EG, Kwasny MJ, Mohr DC (2017). What might get in the way: barriers to the use of apps for depression. Digit Health.

[3] COVID-19 Mental Disorders Collaborators (2021). Global prevalence and burden of depressive and anxiety disorders in 204 countries and territories in 2020 due to the COVID-19 pandemic. Lancet.

[4] Martinez AB, Co M, Lau J, Brown JS (2020). Filipino help-seeking for mental health problems and associated barriers and facilitators: a systematic review. Soc Psychiatry Psychiatr Epidemiol.

[5] Berger T, Hämmerli K, Gubser N, Andersson G, Caspar F (2011). Internet-based treatment of depression: a randomized controlled trial comparing guided with unguided self-help. Cogn Behav Ther.

[6] Mohr DC, Hart SL, Howard I, Julian L, Vella L, Catledge C, Feldman MD (2006). Barriers to psychotherapy among depressed and nondepressed primary care patients. Ann Behav Med.

[7] Mohr DC, Ho J, Duffecy J, Baron KG, Lehman KA, Jin L, Reifler D (2010). Perceived barriers to psychological treatments and their relationship to depression. J Clin Psychol.

[8] Yardley L, Choudhury T, Patrick K, Michie S (2016). Current issues and future directions for research into digital behavior change interventions. Am J Prev Med.

[9] Newby K, Teah G, Cooke R, Li X, Brown K, Salisbury-Finch B, Kwah K, Bartle N, Curtis K, Fulton E, Parsons J, Dusseldorp E, Williams SL (2021). Do automated digital health behaviour change interventions have a positive effect on self-efficacy? A systematic review and meta-analysis. Health Psychol Rev.

[10] Borghouts J, Eikey E, Mark G, de Leon C, Schueller SM, Schneider M, Stadnick N, Zheng K, Mukamel D, Sorkin DH (2021). Barriers to and facilitators of user engagement with digital mental health interventions: systematic review. J Med Internet Res.

[11] Kazdin AE, Rabbitt SM (2013). Novel models for delivering mental health services and reducing the burdens of mental illness. Clin Psychol Sci.

[12] Stringer H (2024). Mental health care is in high demand. Psychologists are leveraging tech and peers to meet the need. Monit Psychol.

[13] Lorence DP, Park H, Fox S (2006). Racial disparities in health information access: resilience of the digital divide. J Med Syst.

[14] Muñoz AO, Camacho E, Torous J (2021). Marketplace and literature review of Spanish language mental health apps. Front Digit Health.

[15] Linardon J, Fuller-Tyszkiewicz M (2020). Attrition and adherence in smartphone-delivered interventions for mental health problems: a systematic and meta-analytic review. J Consult Clin Psychol.

[16] Lipschitz JM, van Boxtel R, Torous J, Firth J, Lebovitz JG, Burdick KE, Hogan TP (2022). Digital mental health interventions for depression: scoping review of user engagement. J Med Internet Res.

[17] Nahum-Shani I, Shaw SD, Carpenter SM, Murphy SA, Yoon C (2022). Engagement in digital interventions. Am Psychol.

[18] Eysenbach G (2005). The law of attrition. J Med Internet Res.

[19] Ji JL, Baee S, Zhang D, Calicho-Mamani CP, Meyer MJ, Funk D, Portnow S, Barnes L, Teachman BA (2021). Multi-session online interpretation bias training for anxiety in a community sample. Behav Res Ther.

[20] Kwon H, Kim HH, An J, Lee JH, Park YR (2021). Lifelog data-based prediction model of digital health care app customer churn: retrospective observational study. J Med Internet Res.

[21] Stieger M, Flückiger C, Rüegger D, Kowatsch T, Roberts BW, Allemand M (2021). Changing personality traits with the help of a digital personality change intervention. Proc Natl Acad Sci U S A.

[22] Tahsin F, Tracy S, Chau E, Harvey S, Loganathan M, McKinstry B, Mercer SW, Nie J, Ramsay T, Thavorn K, Palen T, Sritharan J, Steele Gray C (2021). Exploring the relationship between the usability of a goal-oriented mobile health application and non-usage attrition in patients with multimorbidity: a blended data analysis approach. Digit Health.

[23] Baumel A, Muench F, Edan S, Kane JM (2019). Objective user engagement with mental health apps: systematic search and panel-based usage analysis. J Med Internet Res.

[24] Twomey C, O'Reilly G, Byrne M, Bury M, White A, Kissane S, McMahon A, Clancy N (2014). A randomized controlled trial of the computerized CBT programme, MoodGYM, for public mental health service users waiting for interventions. Br J Clin Psychol.

[25] Harari GM (2020). A process-oriented approach to respecting privacy in the context of mobile phone tracking. Curr Opin Psychol.

[26] Lattie EG, Adkins EC, Winquist N, Stiles-Shields C, Wafford QE, Graham AK (2019). Digital mental health interventions for depression, anxiety, and enhancement of psychological well-being among college students: systematic review. J Med Internet Res.

[27] Miyamoto S, Dharmar M, Fazio S, Tang-Feldman Y, Young HM (2018). mHealth technology and nurse health coaching to improve health in diabetes: protocol for a randomized controlled trial. JMIR Res Protoc.

[28] Schueller SM, Washburn JJ, Price M (2016). Exploring mental health providers' interest in using web and mobile-based tools in their practices. Internet Interv.

[29] Berry N, Bucci S, Lobban F (2017). Use of the internet and mobile phones for self-management of severe mental health problems: qualitative study of staff views. JMIR Ment Health.

[30] Doherty K, Doherty G (2018). Engagement in HCI: conception, theory and measurement. ACM Comput Surv.

[31] Schueller SM, Tomasino KN, Mohr DC (2016). Integrating human support into behavioral intervention technologies: the efficiency model of support. Clin Psychol Sci Pract.

[32] Crafoord MT, Fjell M, Sundberg K, Nilsson M, Langius-Eklöf A (2020). Engagement in an interactive app for symptom self-management during treatment in patients with breast or prostate cancer: mixed methods study. J Med Internet Res.

[33] Linardon J, Shatte A, Tepper H, Fuller-Tyszkiewicz M (2020). A survey study of attitudes toward, and preferences for, e-therapy interventions for eating disorder psychopathology. Int J Eat Disord.

[34] Xie LF, Itzkovitz A, Roy-Fleming A, Da Costa D, Brazeau AS (2020). Understanding self-guided web-based educational interventions for patients with chronic health conditions: systematic review of intervention features and adherence. J Med Internet Res.

[35] Hutchesson MJ, Duncan MJ, Oftedal S, Ashton LM, Oldmeadow C, Kay-Lambkin F, Whatnall MC (2021). Latent class analysis of multiple health risk behaviors among Australian University students and associations with psychological distress. Nutrients.

[36] Chien I, Enrique A, Palacios J, Regan T, Keegan D, Carter D, Tschiatschek S, Nori A, Thieme A, Richards D, Doherty G, Belgrave D (2020). A machine learning approach to understanding patterns of engagement with internet-delivered mental health interventions. JAMA Netw Open.

[37] Kovacs G, Wu Z, Bernstein MS (2018). Rotating online behavior change interventions increases effectiveness but also increases attrition. Proc ACM Hum Comput Interact.

[38] Periáñez Á, Saas A, Guitart A, Magne C (2016). Churn prediction in mobile social games: towards a complete assessment using survival ensembles. Proceedings of the IEEE International Conference on Data Science and Advanced Analytics.

[39] Gersh E, Hallford DJ, Rice SM, Kazantzis N, Gersh H, Gersh B, McCarty CA (2017). Systematic review and meta-analysis of dropout rates in individual psychotherapy for generalized anxiety disorder. J Anxiety Disord.

[40] Goyal S, Morita P, Lewis GF, Yu C, Seto E, Cafazzo JA (2016). The systematic design of a behavioural mobile health application for the self-management of type 2 diabetes. Can J Diabetes.

[41] Pratap A, Neto EC, Snyder P, Stepnowsky C, Elhadad N, Grant D, Mohebbi MH, Mooney S, Suver C, Wilbanks J, Mangravite L, Heagerty PJ, Areán P, Omberg L (2020). Indicators of retention in remote digital health studies: a cross-study evaluation of 100,000 participants. NPJ Digit Med.

[42] Rabbi M, Philyaw Kotov M, Cunningham R, Bonar EE, Nahum-Shani I, Klasnja P, Walton M, Murphy S (2018). Toward increasing engagement in substance use data collection: development of the substance abuse research assistant app and protocol for a microrandomized trial using adolescents and emerging adults. JMIR Res Protoc.

[43] Wong HW, Lo B, Shi J, Hollenberg E, Abi-Jaoude A, Johnson A, Chaim G, Cleverley K, Henderson J, Levinson A, Robb J, Voineskos A, Wiljer D (2021). Postsecondary student engagement with a mental health app and online platform (Thought Spot): qualitative study of user experience. JMIR Ment Health.

[44] Eberle JW, Boukhechba M, Sun J, Zhang D, Funk DH, Barnes LE, Teachman BA (2023). Shifting episodic prediction with online cognitive bias modification: a randomized controlled trial. Clin Psychol Sci.

[45] Murray E, White IR, Varagunam M, Godfrey C, Khadjesari Z, McCambridge J (2013). Attrition revisited: adherence and retention in a web-based alcohol trial. J Med Internet Res.

[46] Nicholas J, Proudfoot J, Parker G, Gillis I, Burckhardt R, Manicavasagar V, Smith M (2010). The ins and outs of an online bipolar education program: a study of program attrition. J Med Internet Res.

[47] Price M, Gros DF, McCauley JL, Gros KS, Ruggiero KJ (2012). Nonuse and dropout attrition for a web-based mental health intervention delivered in a post-disaster context. Psychiatry.

[48] Berry N, Lobban F, Bucci S (2019). A qualitative exploration of service user views about using digital health interventions for self-management in severe mental health problems. BMC Psychiatry.

[49] Cheung K, Ling W, Karr CJ, Weingardt K, Schueller SM, Mohr DC (2018). Evaluation of a recommender app for apps for the treatment of depression and anxiety: an analysis of longitudinal user engagement. J Am Med Inform Assoc.

[50] Kim S, Choi D, Lee E, Rhee W (2017). Churn prediction of mobile and online casual games using play log data. PLoS One.

[51] Yang C, Shi X, Luo J, Han J I know you'll be back: interpretable new user clustering and churn prediction on a mobile social application. arXiv.

[52] Fernandez E, Salem D, Swift JK, Ramtahal N (2015). Meta-analysis of dropout from cognitive behavioral therapy: magnitude, timing, and moderators. J Consult Clin Psychol.

[53] Henkemans OA, Dumay AM, Rogers WA (2011). Personal characteristics and the law of attrition in randomized controlled trials of eHealth services for self-care. Gerontechnology.

[54] Schroé H, Crombez G, de Bourdeaudhuij I, van Dyck D (2022). Investigating when, which, and why users stop using a digital health intervention to promote an active lifestyle: secondary analysis with a focus on health action process approach-based psychological determinants. JMIR Mhealth Uhealth.

[55] Dixon LJ, Linardon J (2020). A systematic review and meta-analysis of dropout rates from dialectical behaviour therapy in randomized controlled trials. Cogn Behav Ther.

[56] De S, Prabu P (2022). Predicting customer churn: a systematic literature review. J Discrete Math Sci Cryptogr.

[57] Jardine J, Nadal C, Robinson S, Enrique A, Hanratty M, Doherty G (2024). Between rhetoric and reality: real-world barriers to uptake and early engagement in digital mental health interventions. ACM Trans Comput Hum Interact.

[58] Tang X, Liu Y, Shah N, Shi X, Mitra P, Wang S (2020). Knowing your FATE: friendship, action and temporal explanations for user engagement prediction on social apps. Proceedings of the 26th ACM SIGKDD International Conference on Knowledge Discovery & Data Mining.

[59] Scherer EA, Ben-Zeev D, Li Z, Kane JM (2017). Analyzing mHealth engagement: joint models for intensively collected user engagement data. JMIR Mhealth Uhealth.

[60] Torous J, Lipschitz J, Ng M, Firth J (2020). Dropout rates in clinical trials of smartphone apps for depressive symptoms: a systematic review and meta-analysis. J Affect Disord.

[61] Kroenke K, Spitzer RL, Williams JB (2003). The patient health questionnaire-2: validity of a two-item depression screener. Med Care.

[62] Salemink E, Kindt M, Rienties H, van den Hout M (2014). Internet-based cognitive bias modification of interpretations in patients with anxiety disorders: a randomised controlled trial. J Behav Ther Exp Psychiatry.

[63] (2010). Readiness ruler. Case Western Reserve University.

[64] MacLeod C, Mathews A (2012). Cognitive bias modification approaches to anxiety. Annu Rev Clin Psychol.

[65] Mathews A, Mackintosh B (2000). Induced emotional interpretation bias and anxiety. J Abnorm Psychol.

[66] Beck AT (1979). Cognitive Therapy and the Emotional Disorders.

[67] Beck AT, Clark DA (1997). An information processing model of anxiety: automatic and strategic processes. Behav Res Ther.

[68] Mathews A, Mackintosh BA (1998). A cognitive model of selective processing in anxiety. Cogn Ther Res.

[69] Yang R, Cui L, Li F, Xiao J, Zhang Q, Oei TP (2017). Effects of cognitive bias modification training via smartphones. Front Psychol.

[70] Fodor LA, Georgescu R, Cuijpers P, Szamoskozi Ş, David D, Furukawa TA, Cristea IA (2020). Efficacy of cognitive bias modification interventions in anxiety and depressive disorders: a systematic review and network meta-analysis. Lancet Psychiatry.

[71] Hallion LS, Ruscio AM (2011). A meta-analysis of the effect of cognitive bias modification on anxiety and depression. Psychol Bull.

[72] Jones EB, Sharpe L (2017). Cognitive bias modification: a review of meta-analyses. J Affect Disord.

[73] Hohensee N, Meyer MJ, Teachman BA (2020). The effect of confidence on dropout rate and outcomes in online cognitive bias modification. J Technol Behav Sci.

[74] Eberle JW, Daniel KE, Baee S, Silverman AL, Lewis E, Baglione AN, Werntz A, French NJ, Ji JL, Hohensee N, Tong X, Huband JM, Boukhechba M, Funk DH, Barnes LE, Teachman BA (2024). Web-based interpretation bias training to reduce anxiety: a sequential, multiple-assignment randomized trial. J Consult Clin Psychol.

[75] MindTrails homepage. MindTrails.

[76] Lovibond PF, Lovibond SH (1995). The structure of negative emotional states: comparison of the depression anxiety stress scales (DASS) with the Beck Depression and Anxiety Inventories. Behav Res Ther.

[77] Namaky N, Glenn JJ, Eberle JW, Teachman BA (2021). Adapting cognitive bias modification to train healthy prospection. Behav Res Ther.

[78] Werntz A, Silverman AL, Behan H, Patel SK, Beltzer M, Boukhechba MO, Barnes L, Teachman BA (2022). Lessons learned: providing supportive accountability in an online anxiety intervention. Behav Ther.

[79] Eberle JW, Baee S, Behan HC, Baglione AN, Boukhechba M, Funk DH, Barnes LE, Teachman BA (2022). TeachmanLab/MT-Data-CalmThinkingStudy: centralized data cleaning for MindTrails calm thinking study. Zenodo.

[80] Blázquez-García A, Conde A, Mori U, Lozano JA (2021). A review on outlier/anomaly detection in time series data. ACM Comput Surv.

[81] Mowbray FI, Fox-Wasylyshyn SM, El-Masri MM (2019). Univariate outliers: a conceptual overview for the nurse researcher. Can J Nurs Res.

[82] Goldberg SB, Bolt DM, Davidson RJ (2021). Data missing not at random in mobile health research: assessment of the problem and a case for sensitivity analyses. J Med Internet Res.

[83] Pujianto U, Murti DM, Wibawa AP, Akbar MI (2019). K-nearest neighbor (K-NN) based missing data imputation. Proceedings of the 5th International Conference on Science in Information Technology.

[84] Kim KY, Kim BJ, Yi GS (2004). Reuse of imputed data in microarray analysis increases imputation efficiency. BMC Bioinformatics.

[85] Borkovec TD, Nau SD (1972). Credibility of analogue therapy rationales. J Behav Ther Exp Psychiatry.

[86] Cheung F, Lucas RE (2014). Assessing the validity of single-item life satisfaction measures: results from three large samples. Qual Life Res.

[87] Wolpe J (1969). The practice of behavior therapy.

[88] Clark DM, Salkovskis PM, Öst LG, Breitholtz E, Koehler KA, Westling BE, Jeavons A, Gelder M (1997). Misinterpretation of body sensations in panic disorder. J Consult Clin Psychol.

[89] Norman SB, Cissell SH, Means-Christensen AJ, Stein MB (2006). Development and validation of an overall anxiety severity and impairment scale (OASIS). Depress Anxiety.

[90] Kroenke K, Spitzer RL, Williams JB, Monahan PO, Löwe B (2007). Anxiety disorders in primary care: prevalence, impairment, comorbidity, and detection. Ann Intern Med.

[91] Collins RL, Parks GA, Marlatt GA (1985). Social determinants of alcohol consumption: the effects of social interaction and model status on the self-administration of alcohol. J Consult Clin Psychol.

[92] Ersner-Hershfield H, Garton MT, Ballard K, Samanez-Larkin GR, Knutson B (2023). Don’t stop thinking about tomorrow: individual differences in future self-continuity account for saving. Judgm Decis Mak.

[93] Dennis JP, Vander Wal JS (2009). The cognitive flexibility inventory: instrument development and estimates of reliability and validity. Cogn Ther Res.

[94] Francis AW, Dawson DL, Golijani-Moghaddam N (2016). The development and validation of the comprehensive assessment of acceptance and commitment therapy processes (CompACT). J Contextual Behav Sci.

[95] Gross JJ, John OP (2003). Individual differences in two emotion regulation processes: implications for affect, relationships, and well-being. J Pers Soc Psychol.

[96] Carleton RN, Norton MP, Asmundson GJ (2007). Fearing the unknown: a short version of the intolerance of uncertainty scale. J Anxiety Disord.

[97] Chen G, Gully SM, Eden D (2001). Validation of a new general self-efficacy scale. Organ Res Methods.

[98] Dweck CS (2007). Mindset: The New Psychology of Success.

[99] Scheier MF, Carver CS, Bridges MW (1994). Distinguishing optimism from neuroticism (and trait anxiety, self-mastery, and self-esteem): a reevaluation of the life orientation test. J Pers Soc Psychol.

[100] Flanagan JC (1978). A research approach to improving our quality of life. Am Psychol.

[101] Chawla NV, Bowyer KW, Hall LO, Kegelmeyer WP (2002). SMOTE: synthetic minority over-sampling technique. J Artif Intell Res.

[102] Elreedy D, Atiya AF (2019). A comprehensive analysis of synthetic minority oversampling technique (SMOTE) for handling class imbalance. Inf Sci.

[103] Bellinger C, Sharma S, Japkowicz N (2012). One-class versus binary classification: which and when?. Proceedings of the 11th International Conference on Machine Learning and Applications.

[104] Kumari R, Srivastava SK (2017). Machine learning: a review on binary classification. Int J Comput Appl.

[105] Hosmer DW Jr, Lemeshow S, Sturdivant RX (2013). Applied Logistic Regression.

[106] Noble WS (2006). What is a support vector machine?. Nat Biotechnol.

[107] Chen T (2014). Introduction to boosted trees. University of Washington.

[108] Chen T, He T, Benesty M, Khotilovich V, Tang Y, Cho H, Chen K, Mitchell R, Cano I, Zhou T, Li M, Xie J, Lin M, Geng Y, Li Y, Yuan J (2024). XGBoost: extreme gradient boosting. The Comprehensive R Archive Network.

[109] Johnson R, Zhang T (2014). Learning nonlinear functions using regularized greedy forest. IEEE Trans Pattern Anal Mach Intell.

[110] Gardner MW, Dorling SR (1998). Artificial neural networks (the multilayer perceptron)—a review of applications in the atmospheric sciences. Atmos Environ.

[111] Bergstra J, Yamins D, Cox DD (2013). Hyperopt: a python library for optimizing the hyperparameters of machine learning algorithms. Proceedings of the 12th Python in Science Conference.

[112] Nembrini S, König IR, Wright MN (2018). The revival of the Gini importance?. Bioinformatics.

[113] van Bronswijk SC, DeRubeis RJ, Lemmens LH, Peeters FP, Keefe JR, Cohen ZD, Huibers MJ (2021). Precision medicine for long-term depression outcomes using the Personalized Advantage Index approach: cognitive therapy or interpersonal psychotherapy?. Psychol Med.

[114] Tsetsi E, Rains SA (2017). Smartphone internet access and use: extending the digital divide and usage gap. Mob Media Commun.

[115] Yu R, Lee H, Kizilcec RF (2021). Should college dropout prediction models include protected attributes?. Proceedings of the Eighth ACM Conference on Learning @ Scale.

[116] Karimi-Haghighi M, Castillo C, Hernandez-Leo D, Oliver VM Predicting early dropout: calibration and algorithmic fairness considerations. arXiv.

[117] Morgan C, Mason E, Newby JM, Mahoney AE, Hobbs MJ, McAloon J, Andrews G (2017). The effectiveness of unguided internet cognitive behavioural therapy for mixed anxiety and depression. Internet Interv.

